# Capacity management of nursing staff as a vehicle for organizational improvement

**DOI:** 10.1186/1472-6963-7-196

**Published:** 2007-11-30

**Authors:** Sylvia G Elkhuizen, Gert Bor, Marjolein Smeenk, Niek S Klazinga, Piet JM Bakker

**Affiliations:** 1 Department of Innovation and Process Management, Academic Medical Center/University of Amsterdam, Amsterdam, The Netherlands; 2 Department of Social Medicine, Academic Medical Center/University of Amsterdam, Amsterdam, The Netherlands

## Abstract

**Background:**

Capacity management systems create insight into required resources like staff and equipment. For inpatient hospital care, capacity management requires information on beds and nursing staff capacity, on a daily as well as annual basis. This paper presents a comprehensive capacity model that gives insight into required nursing staff capacity and opportunities to improve capacity utilization on a ward level.

**Methods:**

A capacity model was developed to calculate required nursing staff capacity. The model used historical bed utilization, nurse-patient ratios, and parameters concerning contract hours to calculate beds and nursing staff needed per shift and the number of nurses needed on an annual basis in a ward. The model was applied to three different capacity management problems on three separate groups of hospital wards. The problems entailed operational, tactical, and strategic management issues: optimizing working processes on pediatric wards, predicting the consequences of reducing length of stay on nursing staff required on a cardiology ward, and calculating the nursing staff consequences of merging two internal medicine wards.

**Results:**

It was possible to build a model based on easily available data that calculate the nursing staff capacity needed daily and annually and that accommodate organizational improvements. Organizational improvement processes were initiated in three different groups of wards. For two pediatric wards, the most important improvements were found to be improving working processes so that the agreed nurse-patient ratios could be attained. In the second case, for a cardiology ward, what-if analyses with the model showed that workload could be substantially lowered by reducing length of stay. The third case demonstrated the possible savings in capacity that could be achieved by merging two small internal medicine wards.

**Conclusion:**

A comprehensive capacity model was developed and successfully applied to support capacity decisions on operational, tactical, and strategic levels. It appeared to be a useful tool for supporting discussions between wards and hospital management by giving objective and quantitative insight into staff and bed requirements. Moreover, the model was applied to initiate organizational improvements, which resulted in more efficient capacity utilization.

## Background

Market changes, labor shortages, and the introduction of a form of activity-based costing (diagnosis treatment combination (DTC) financing policy) in the Netherlands have provided an impetus for hospitals to reorganize care processes to improve efficiency. Establishing a form of capacity management with regard to agreed service levels of quality of care is essential to gaining insight into the balance between available and required resources, like staff and equipment. For inpatient care facilities in a hospital, this requires information on bed capacity and nursing staff capacity, on a daily as well as annual basis. Quantitative models can be used to calculate capacity needs for different planning purposes and for short, medium and long term planning issues. Although several useful models are described in the international literature [1-3] many of them are difficult to apply in practice because they require a great deal of data and clerical work [1].

To be able to apply capacity management in practice, models must fulfill different functions: "annual staff planning," "roster scheduling support," and "daily assignment of nurses to wards [4-6]." In addition, "strategic decisions" are sometimes mentioned as a separate planning level [3,7,8]. Models based on mathematical optimization techniques from operations research are generally focused on short-term scheduling [3,9-11]. Models that do integrate different planning horizons (daily, periodical (1–2 months), and annual) are for example described by Abernathy *et al.*[7] and Wright *et al.*[11]. These models contain connected models for periodical staff planning and daily scheduling. However, models incorporating operational planning issues with tactical and strategic decisions or operational scheduling support with annual staff planning were not found in the literature. For accurate capacity management, operational planning issues (like the number of nurses on each shift) should be handled together with tactical planning issues (like annual nurse staffing).

In general, capacity models are aimed at calculating the number of nurses needed, whereas capacity management models should ideally also give insight into opportunities for improving capacity usage. In the Academic Medical Center (AMC) in Amsterdam, the Netherlands, we aimed to create a capacity model that could be used for calculating the number of nurses needed in a hospital ward and that would also provide a projection on how to utilize staff more efficiently. This model has to support decisions on three levels:

- At **operational level**, the most relevant topic is whether the organization of nursing activities is such that appropriate and efficient care can be provided.

- Questions at a **tactical decision **level that should be supported with a capacity management model concern capacity consequences of changes in length of stay (LOS), number of admissions, and patient acuity.

- Relevant **strategic issues **are the optimal number of beds per ward and potential savings in nursing staff capacity by using small flexible nursing pools shared by related wards.

Ideally, capacity management would support discussion between management and staff on all these issues and promote improvements.

This paper describes a comprehensive capacity management model that gives insight into:

- the number of nurses needed for a hospital ward, and

- opportunities to improve capacity utilization on a ward.

Three cases will be used to demonstrate how this model can be applied to support issues at operational, tactical, and strategic levels.

## Methods

**Figure 1:**
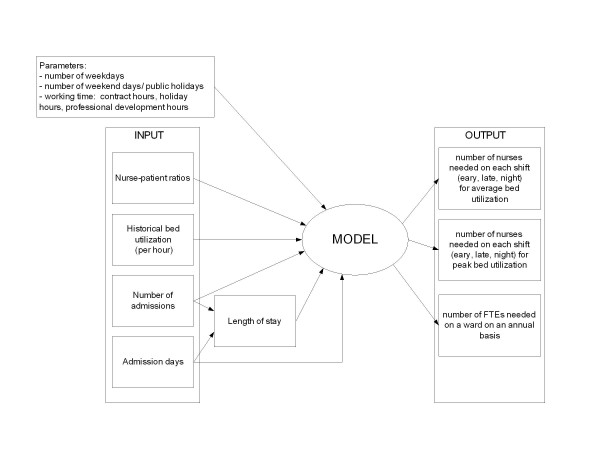
Outline of model.

An outline of the model is given in Figure 1. In this paragraph, first the core data will be described, followed by a paragraph about the model and finishing with the description of how the model is applied in three case studies.

### Core data

The basis of the model is formed by historical bed utilization and nurse-patient ratios. Historical bed utilization gives the number of beds used on a daily basis and was available for all wards on an hourly basis from January 2005. For an inpatient department, quality of nursing care is directly related to direct nursing hours per patient [12]. Available direct nursing hours per patient depends on nurse-patient ratios and indirect activities. So, an appropriate nurse-patient ratio can be determined by using a standard for both indirect activities and direct nursing hours. For example, when each patient needs an average of 1.2 hours of direct care and a nurse spends two hours on indirect activities during each shift, each nurse can handle five patients in an 8 hour shift – the nurse-patient ratio can then be set at 1:5. During evening and night shifts, fewer care hours per patient are needed, so ratios can be set at a somewhat higher level (more patients per nurse), for example 1:6 or 1:8. Guidelines for nurse-patient ratios can be derived from the international literature. In two countries – USA (California) and Australia (Victoria) – legal minimums for nurse-patient ratios were set for general medical and surgical wards [13]. These minimum ratios were 1:5 in California and vary between 1:4 and 1:6 in Victoria for day shifts. Research on the relationship between patient outcomes and nurse-patient ratios has shown that the higher the ratios, the more adverse events occurred [14,15]. Because of this, we advocate setting nurse-patient ratios at around 1:4–5 for early shifts, 1:6–7 for late shifts, and 1:8–10 for night shifts. The ratios applied in the AMC differ somewhat between wards.

To transform direct nursing care hours into the full-time equivalents (FTEs) needed annually, data were used from agreements on working conditions for all university hospitals in the Netherlands (such as contract hours, holiday hours, and time allotted for professional development).

To perform tactical what-if analyses (changing parameters, and comparing effects) the number of admissions and total number of admission days was included. Average LOS can be calculated by dividing total admission days by the number of admissions.

### Model

Operational output of the model is the number of nurses that need to be scheduled for each shift. For the tactical level, the model provides the number of FTEs needed annually for direct care on a ward. To be able to analyze the strategic issue of the possibilities for flexible nursing staff, the number of nurses needed for average bed utilization and the extra number needed in peak periods are separated. The strategic issue of ward size can be analyzed by comparing model results for separate wards with model results using aggregated data for several wards.

The calculated model results can be compared with the current number of nurses. To make a fair comparison, only nurses available for direct patient care were included. Additional staff, as the ward manager, are not included in the model and in the comparison between model results and actual situation. This means that differences were presumed to be rooted in a variety of possible causes at different decision levels:

#### Operational issues

- Working methods are not efficient enough to meet the agreed nurse-patient ratio with current nursing staff capacity. An effective way to explore this further is to have a relative outsider observe the working processes, for example someone from a comparable department that does meet the ratios.

- Sick leave (including maternity leave) in the ward differs substantially from the standard 4% level.

#### Tactical issues

- Applied nurse-patient ratios are not in accordance with average patient acuity levels on the ward. To investigate this in more detail for a specific ward, acuity levels can be analyzed for a sample of patients.

- Bed utilization is considerable with respect to current production. This could be caused by a lack of efficient admission and discharge processes, which lead to unnecessarily long LOS.

- The ward has nursing staff capacity shortage or surplus.

#### Strategic issues

- Wards are too small, which results in efficiency losses, especially during night shifts.

- A great deal of fluctuation in bed utilization can lead to inefficiency. Introducing a flexible nursing pool could be an option.

All input parameters can be used for what-if analyses. These analyses were checked by comparing the results using bed utilization in 2006 as input with the results using historical bed utilization for 2005, combined with changes in admissions LOS from 2005 to 2006.

### Model application

The model was applied to three different capacity management problems on three separate groups of hospital wards. The problems entailed operational, tactical, and strategic management issues: optimizing working processes on pediatric wards, predicting consequences of LOS reduction on required nursing staff on a cardiology ward, and calculating the nursing staff consequences of merging two internal medicine wards. Nurse-patient ratios were agreed upon. For historical bed utilization, the number of beds and nurses needed for every shift and on an annual basis were calculated. What-if analyses were performed and further examinations of working processes were carried out.

## Results

### Core data

Nurse-patient ratios were standard set on 1:4 for day shift, 1:6 for evening shifts and 1:8 for night shifts. Actual applied ratios in different analyses were agreed between hospital management and ward staff. The model uses historical bed utilization, which is recorded on an hourly basis for each ward. The model uses a number of parameters, which were derived from agreements on working conditions for all university hospitals in the Netherlands. Table 1 gives these parameters along with the values used. All these values in the model can be easily changed.

**Table 1:** Parameters and values of the model.

Contractual annual working hours per FTE		1872.0
Holiday hours	9%	168.5
Extra holiday hours for employees > age 45	0.2%	3.7
Compensation for official holidays	3.5%	65.5
Professional development	2%	37.4
Average sick leave	4%	74.9
Available annual working hours per FTE		1521.9

The number of admissions and total number of admission days were extracted from our hospital management information system. The source of the number of admission numbers and days was the same as the source of the hourly bed utilization. Therefore, the total number of admissions had resulted into the recorded hourly bed utilization. Average LOS can be calculated by dividing total admission days by the number of admissions.

### Model

**Figure 2:**
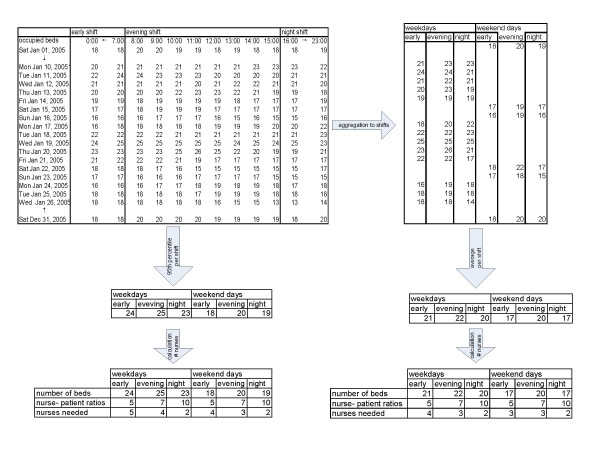
Using historical bed utilization to calculate the number of nurses needed per shift.

The hourly historical bed utilization is aggregated into the maximum number of beds used during each shift. For example, when from 8 to 11 a.m. 18 beds were occupied, from 11 a.m. to 1 p.m. 19 beds and from 2 pm to 3 pm 17 beds, the 'bed utilization' for this dayshift was set on the maximum of 19 beds. Subsequently the model calculates the average for six different shifts: weekdays and weekend days, each separated into early, evening, and night shifts. The early shifts use the hourly measures from 8 a.m. until 3 p.m., the evening shifts from 4 p.m. until 11 p.m., and the night shifts from midnight until 7 a.m.. So, all 24 measures were equally divided over the three daily shifts. Using the hourly bed utilization, the 95^th ^percentile is also calculated for each shift. This is defined as the number of beds for which occupancy is equal to or less than this in 95% of all cases. By dividing the number of beds by the nurse-patient ratio, the number of nurses for each shift is calculated for both the average and the 95^th ^percentile scenario. The precondition for this is that each shift has a minimum of two nurses. All steps used in the calculation are presented in Figure 2.

With these calculations, we created two scenarios: the 'average' scenario and the 95^th ^percentile scenario. The annual number of nursing hours is calculated for both scenarios (average and 95th percentile) using the number of weekdays (255), the number of weekend and official holidays (110), and the shift durations for nurses for the early shift (8 hours), evening shift (8 hours), and night shift (9 hours). The number of FTE nurses for a ward is calculated by dividing this by annual availability (see Table 1). This is the number of FTEs needed for direct patient care. The 95^th ^percentile scenario gives the number of nurses needed for patient care. This implies that 5% of the time, nurses have to handle a few more beds than the ratio indicates which corresponds with current practice. Providing all wards with enough nurses in all cases (100%) would result in a great deal of overcapacity. The average scenario is calculated to gain some understanding into the difference between average and peak load in that specific ward. Another idea is that the number of additional nurses needed between the average and the 95% scenario could possibly be shared by similar wards. This would mean that a small pool of nurses could be formed to work on two or three related wards, where the nature of patient care is comparable. Because in general, nurses are not contracted to work on a specific ward, changing between wards should be possible. Perhaps, not all employees are willing to change between wards. However, for experienced nurses it can offer an opportunity to broaden their work field and flexibility can be rewarded to motivate employees [16].

Apart from the model, the number of overhead and supporting functions have to be determined. These are independent of bed utilization. For analysis purposes, historical production data with regard to number of admissions and patient days are added into the model.

The what-if analyses were checked for an internal medicine ward; results are given in Table 2. First, we applied the model with historical bed utilization data for 2005. Next, a what-if analysis was performed using the growth for this ward in 2006. This resulted in a change in the number of admissions, from 1,290 in 2005 to 1,345 in 2006 (+4%), and the LOS increased from 6.5 in 2005 to 6.7. This led to suggesting the addition of one nurse to the early weekday shifts while keeping all other shifts unchanged. The annual number of FTEs have to increase from 25.7 to 27. This corresponds exactly to the model results using historical bed utilization for 2006.

**Table 2:** Comparison of what-if analysis applied to the 2005 model with growth for 2006 and model results for 2006.

Model results	Weekdays			Weekend/holidays			FTEs
	Early	Evening	Night	Early	Evening	Night	

With historical data 2005	6	4	3	6	4	3	25.7
2005 +what-if scenario with growth for 2006	7	4	3	6	4	3	27.0
With historical data 2006	7	4	3	6	4	3	27.0

### Model application

#### Case: Operational changes in pediatric wards

Five pediatric wards lacked transparency in capacity requirements. Also, their staff planning was based on a situation that arose over time, with many inexplicable differences between wards. The model was applied to evaluate current numbers of nurses per shift in order to provide a foundation for the need for more efficient working practices, and to calculate budgets for nurses. The model results for the pediatric wards are presented in Table 3. The model gives the number of nurses that have to be planned for each shift and the number of nurses needed annually for this planning. For all wards except one (teenagers), the applied nurse-patient ratios were low compared with ratios in other wards. In this case, most attention was given to investigating what working processes could be improved to increase nurse-patient ratios. The main conclusions were that even with the relatively low ratios there is a surplus of nurses and that inexplicable differences between the wards must be corrected. For the five pediatric wards, a total of 5.1 FTE can be saved annually. With a yearly cost per FTE of €45.000 (including all costs and bonuses), this leads to a saving of more than 200.000. Working processes were observed over several weeks in two wards (infants and children) that did not meet the agreed nurse-patient ratios. For the observations, structured sheets were used on which the observers noted activities and contacts of the observed employees. We observed not only nurses, but also receptionists and physicians, since they also influence nurses efficiency. Each function was observed several days by different observers. Qualitative and quantitative observations were collected and put together to find the bottlenecks. The main causes for inefficiency appeared to be disorganized physician rounds and uncertainty about admission and discharge planning. For both wards, suggestions for improvement were made to the ward management. The benefit of using the model before observing bottlenecks was that the wards presenting the greatest challenges could be selected, quantitative insight could be provided for inefficiencies, and other possible causes could be excluded. The model results were incorporated into the budgets for 2007.

**Table 3:** Model results for five pediatric wards.

			Weekdays			Weekend/holidays			No of nurses according to model (yearly FTE)	Actual number of nurses in 2005 (yearly FTE)	Difference
Ward	Available beds		Early	Evening	Night	Early	Evening	Night			

Infants (0 years)	19	Number of beds	16	16	16	15	15	15	22.6	26.7	-4.1
		Nurse-patient ratios	3.5	4.5	5.5	3.5	4.5	5.5			
		Nurses needed	5	4	3	4	3	3			
Children (1–9 years)	24	Number of beds	21	20	20	14	14	15	23.2	25.5	-2.2
		Nurse-patient ratios	3.5	5	6.5	3.5	5	6.5			
		Nurses needed	6	4	3	4	3	2			
Teenagers (10–17) years	24	Number of beds	23	22	22	19	19	20	24.5	22.4	2.1
		Nurse-patient ratios	4	6	8	4	6	8			
		Nurses needed	6	4	3	5	3	3			
Oncolgy	13–19	Number of beds	18	15	15	14	13	13	26.4	26.9	-0.5
		Nurse-patient ratios	2.5	4	5	2.5	4	5			
		Nurses needed	7	4	3	6	3	3			
Surgery	8	Number of beds	8	8	8	8	7	8	9.8	10.2	-0.4
		Nurse-patient ratios	3.5	4.5	5.5	3.5	4.5	5.5			
		Nurses needed	2	2	1	2	2	1			

#### Case: Tactical decisions on a cardiology ward

For cardiology, the model was applied to gain insight into the possibilities for decreasing the experienced workload. The focus here was to decrease nurse-patient ratios together with reducing the LOS, which are both tactical decision levels. The model results for the cardiology ward are presented in Table 4. The first conclusion was that the available nurses exceeded the model results. However, the nurse-patient ratios this ward adopted seemed to be high for their patients. Re-applying the model with the ratios 1:4, 1:6, and 1:8 for early, evening, and night shifts revealed that 29.8 nurses were needed. So, the experienced workload was confirmed by the model results. With the nurse-patient ratios used (which seemed appropriate for the patient mix in this ward), a shortage of nurses appeared. However, in this case, production data revealed a relatively long average LOS of 12 days. What-if scenarios showed that a LOS reduction of two calendar days led to nurse requirements of 25.1 FTEs, which meets current capacity. So, in this case the same production in numbers of admissions could be done with current capacity and reduced workload by LOS reduction. A main contribution to LOS reduction was made by starting earlier with preparation for discharge. When the department should decide to use the free fallen capacity by increasing the number of admissions, again a shortage of nurses will result, which could be quantified by a what-if analyses concerning production numbers. However, the department had no intention to increase their admissions.

**Table 4:** Model results for cardiology.

	Weekdays			Weekend/holidays					
	Early	Evening	Night	Early	Evening	Night	No of nurses according to model	Actual number of nurses in 2005	Difference

Number of beds*	29	28	28	24	26	24	22.4	25.8	-3.4
Nurse-patient ratios	5	7.5	15	5	7.5	15			
Nurses needed	7	5	4	6	4	3			

*Available beds: 32

#### Case: Strategic improvements in internal medicine wards

The model was applied to two small internal medicine wards to investigate the possible savings of merging small wards. A drawback of small wards is that fluctuations in utilization are relatively large and require more capacity. Moreover, due to the requirement that a minimum of two nurses be available during each shift, the capacity needed can become unrelated to bed utilization and ratios. An analysis was made of possible savings resulting from merging these small wards. The results of this are given in Table 5. This merging could reduce required staff by 3.2 FTEs. This leads to a 144000 euros cost saving annually. However, the internal medicine department is investigating the possibility of concentrating nighttime admissions in one ward, thereby increasing the ratio to 1:10 in night shifts on other wards. Merging these two wards would then save one nurse each night, or 2.2 FTEs annually. Moreover, merging small wards saves on overhead staff functions such as department staff and receptionists.

**Table 5:** Model results for two internal medicine wards.

Ward	Available regular beds		Weekdays			Weekend/holidays			No of nurses according to model
			Early	Evening	Night	Early	Evening	Night	

Internal Medicine	18	Number of beds	18	17	17	16	16	16	19.1
(F6ZU)		Nurse-patient ratios	4	6	8	4	6	8	
		Nurses needed	5	3	2	4	3	2	
Internal Medicine	16	Number of beds	16	15	15	15	15	15	17.7
(F7NO)		Nurse-patient ratios	4	6	8	4	6	8	
		Nurses needed	4	3	2	4	3	2	
Total	34	Number of beds	34	32	32	31	31	31	36.8
		Nurse-patient ratios	4	6	8	4	6	8	
		Nurses needed	9	6	4	8	6	4	
One ward	34	Number of beds	33	32	31	31	31	31	33.6
F6ZU+ F7NO		Nurse-patient ratios	4	6	8	4	6	8	
		Nurses needed	8	5	4	8	5	4	
Difference			1	1		1	1		3.2

## Discussion

A comprehensive model for capacity planning was developed. In many Dutch hospitals, as is the case in other countries, nurse staffing is historically-based. The model offers the opportunity to calculate staffing requirements for wards based, on recent ward-specific data. With the model, required capacity can be calculated on an annual basis and for each shift. Decision support for operational, tactical, and strategic levels is possible with the model: efficiency of working processes can be evaluated, wards can be compared, capacity needed can be compared with staff budgets, consequences of changing LOS and production targets (number of admissions) can be calculated, and ward sizes can be evaluated in terms of required nursing staff capacity. It appeared to be a useful tool for supporting discussions regarding capacity management issues between wards and hospital management by giving objective and quantitative insight into staff and bed requirements.

In three cases, it was shown that the model offered different ideas for the best way to improve capacity utilization. For pediatrics, opportunities for improvement were found at the operational level in the organization of working processes. With more efficient physician rounds and standardized admission and discharge procedures, the nurse-patient ratios agreed with the wards were attainable. This calculated annual nursing staff was adopted in the 2007 budget, and the existing inexplicable differences between wards were removed. For the cardiology department, the key to reducing workload was to reduce LOS, by keeping the same number of admissions. Finally, for two internal medicine wards, possible savings were calculated in nursing staff capacity by merging the wards, a strategic level decision. In all cases, the model was applied as part of a larger project.

The model goes beyond efficiency and cost reduction [17]. It offers insight into opportunities to improve working processes and reduce workload, which makes the nurses' work easier. Employing an adequate number of nurses is beneficial for patients and for nurses themselves [18,19]. One of the strong points of the model is the use of historical utilization data rather than available beds. This means both annual production and fluctuation in utilization are taken into account and provides a more fair comparison between wards and between current and calculated numbers of staff. In the AMC, the hourly bed utilization data are regularly recorded and are easily accessible for use in the model. The model is also applicable with less detailed data like maximum or average bed utilization per shift. For the observation at the pediatric wards, structured observations were used. Limitation was that no inter-observer consistency was calculated.

The model has not the aim to replace integrated hospital and patient planning methods (see for example [20,21]). Our model had the aim to support capacity management decisions on ward level, by calculating staffing needs for different planning levels. Apart form hospital wide planning, a lot of capacity improvements can be attained by carefully analyzing capacity utilization on single hospital units. With our model alternative possibilities for improvement can be determined by what-if analyses.

Daily staff scheduling is not incorporated in the model. For the scheduling of nurses, several solutions are available in literature (see for example [11]). Other models concern the short term adaptation of schedules (see for example [22,23]). Our model adds a possibility to provide a quick insight in capacity needs and opportunities to improve capacity usage.

The model could potentially be criticized for not considering patient acuity. In the literature, there is some criticism of the application of nurse-patient ratios [24,25]. However, alternative models are much more complicated and many aspects of patient acuity and organization of care can be incorporated into nurse-patient ratios. Differences in patient care needs between wards can be taken into account by adjusting the nurse-patient ratios. We used the US and Australian ratios as guideline in our discussions with department staff. However, in all case studies, we applied ward-specific ratios that take the Dutch situation and ward specific patient needs into account. For example, as can be seen in Table 3, we applied different ratios for each pediatric ward. These ratios were set by mutual agreement between hospital management and department staff of the pediatric wards. For the levels of analysis in this model, it is sufficient to apply an average nurse patient ratio per ward. This will be sufficient to calculate the number of nurses needed in each shift and yearly, and to perform the what-if analyses. To measure the acuity for patients on a specific ward for a period of time the AUKUH Acuity/Dependency tool developed for the NHS may be useful [26]. This tool supports the measurement of acuity and dependency across a range of wards and specialties. For detailed adaptation of daily nursing staff, daily measurement of patient acuity could be required, due to variety in admitted patients at a moment [22,23,27]. However, in our cases, we checked the acuity mix of patients to investigate the stability of the number of needed nurses in each shift. The patient mix for a sample of weekdays was analyzed for the pediatric and cardiology wards. Each day on each ward there appeared to be a balanced patient mix of mostly "average" patients, a few patients with lower care needs, and even fewer patients with relatively high care needs. Therefore, we are convinced that the calculated number of nurses for each shift is appropriate for safe and adequate patient care. When this is not the case, for example for very small wards, daily adjustment of staffing numbers may be needed. The smaller the number of patients, the larger the variety in number of patients as well as acuity mix will be [28]. These short term adaptation are, however, beyond the scope of this model, that is developed for planning purposes on operational, tactical and strategic level, and not for daily scheduling.

Nursing mix by training levels is not an explicit part of the model. The literature shows that deploying highly educated nurses is cost-effective compared with lower educated nurses [3,29,30]. Due to increasing patient complexity, there is an increasing need for Registered Nurses compared to lower educated staff [3,17]. The model calculates the total number of nurses needed during each shift. In Dutch hospitals, the majority of those in nursing teams are certified nurses. It was assumed that in each shift a sufficient number of qualified nurses would be scheduled. The actual allocation of nurses, and therefore the skill-mix available in a shift is more a scheduling issue which is outside the scope of the model. A future model extension could be the incorporation of different skills in the staffing needs to overcome this limitation.

Two research areas were explored to extend research with the model. First, the application of flexible resource pools will be studied. A model option is to separate average capacity needed per shift and the extra capacity needed in peak periods. This peak capacity could possibly be shared by two or more related wards. Second, the model appears to be suitable for benchmarking resource utilization between comparable wards in different hospitals. Benchmarking projects were started with two other academic hospitals in the Netherlands.

## Conclusion

A comprehensive model could be developed that covers both capacity planning for nursing staff and improving capacity utilization in hospital wards. The model was applied successfully in supporting capacity decisions on operational, tactical, and strategic levels. For two pediatric wards, improvements were made by improving working processes so that the agreed nurse-patient ratios could be attained. In a second case, for a cardiology ward, what-if analyses with the model showed that the workload could be substantially decreased by reducing LOS. The third case demonstrated possible savings in capacity by merging two small internal medicine wards. The model appeared to be useful in calculating capacity needed and in initiating organizational improvements, resulting in more efficient capacity utilization.

## Competing interests

The author(s) declare that they have no competing interests.

## Authors' contributions

SG developed and applied the model, collected the data and wrote the manuscript. GB and MS participated in model development, data collection and model application. NS participated in writing the manuscript. PJB participated in model development and application and in writing the manuscript. All authors approved the final version of the manuscript.

## Pre-publication history

The pre-publication history for this paper can be accessed here:


